# Microbial carbon use efficiency predicted from genome-scale metabolic models

**DOI:** 10.1038/s41467-019-11488-z

**Published:** 2019-08-08

**Authors:** Mustafa Saifuddin, Jennifer M. Bhatnagar, Daniel Segrè, Adrien C. Finzi

**Affiliations:** Boston University, Boston, MA 02215 USA

**Keywords:** Carbon cycle, Carbon cycle, Microbial ecology, Biochemical networks

## Abstract

Respiration by soil bacteria and fungi is one of the largest fluxes of carbon (C) from the land surface. Although this flux is a direct product of microbial metabolism, controls over metabolism and their responses to global change are a major uncertainty in the global C cycle. Here, we explore an in silico approach to predict bacterial C-use efficiency (CUE) for over 200 species using genome-specific constraint-based metabolic modeling. We find that potential CUE averages 0.62 ± 0.17 with a range of 0.22 to 0.98 across taxa and phylogenetic structuring at the subphylum levels. Potential CUE is negatively correlated with genome size, while taxa with larger genomes are able to access a wider variety of C substrates. Incorporating the range of CUE values reported here into a next-generation model of soil biogeochemistry suggests that these differences in physiology across microbial taxa can feed back on soil-C cycling.

## Abstract (web summary)

Microbial respiration releases carbon from the soil. Here, the authors estimate bacterial carbon use efficiency in soils for over 200 species using constraint-based modeling, incorporate the values into an ecosystem model, and find that shifts in community composition may impact carbon storage.

## Introduction

Soil respiration is one of the largest exchanges of carbon (C) from the land surface to the atmosphere, releasing an estimated amount of 98 ± 12 Pg C/year from soil as CO_2_^1,2^. Heterotrophic respiration (R_H_) by soil bacteria and fungi can account for a large proportion of total global soil respiration (35–69 Pg C/year^3,4^). Although this flux is a direct product of microbial metabolism, controls over physiology and their responses to global change are a major uncertainty in the global C cycle^5,6^.

Soil respiration rates are influenced by microbial physiology because cellular metabolism dictates what fraction of the C consumed by soil bacteria and fungi is allocated to respiration, biomass, extracellular enzyme production, and other metabolic functions. Thus, the partitioning of microbial C can have a direct impact on the global C cycle^5^. Variations in C partitioning among microbial taxa is poorly characterized, yet critical to understanding relationships between physiology, community composition, and soil C cycling.

Carbon use efficiency (CUE) measures the partitioning of C between microbial biomass and respiration^7^. Empirical estimates of microbial CUE range from near zero to over 0.8^8,9^. Most biogeochemical models use a fixed value selected between 0.15 and 0.6, typically without careful parameterization^8^. Some of the variation observed in CUE may be attributed to the sensitivity of CUE to abiotic factors such as temperature and pH^8,10^. However, an additional and often neglected source of variation in CUE may be due to physiological differences between soil microbial groups and their differential capacities for accessing particular substrate types^7,11,12^. This single parameter in microbial biogeochemistry models has direct impacts on estimates of greenhouse gas emissions and terrestrial C storage^5,6^, and the sustainability of bioenergy cropping systems^13^, making it necessary to survey how CUE varies both among taxa and across substrate types.

Characterizing microbial C metabolism is particularly important in the context of global change, which may alter the structure and activity of microbial communities and their access to substrates^14^. Previous work on functional and physiological variability suggests that defining bacterial taxa along a spectrum of copiotrophy (fast-growing, adapted to high substrate availability) to oligotrophy (slow-growing, adapted to limiting resource concentrations) may be one useful approach for understanding how groups respond to changes in temperature and resource availability^15,16^. Classification schemes based on trophic strategy may be useful from a biogeochemical perspective if differences in growth strategies correspond to variation in CUE. For example, copiotrophs are hypothesized to show lower CUE than oligotrophs^12,17^, and this could potentially alter the CUE of microbial communities observed to shift toward a greater proportion of copiotrophic bacteria in response to global change manipulations that increase substrate availability, such as soil warming^18,19^.

Observations from global change experiments and genome-based estimates of minimal generation times have shown some support for the classification of particular phyla, as oligotrophic or copiotrophic^14^. However, these phylum-level classifications are not consistent across studies^20,21^. An improved understanding of the phylogenetic structure of biogeochemically relevant traits is needed to identify how microbial community structure impacts C cycling^22,23^. Many functional genes show strong conservation within prokaryotes, leading to the possibility for strong phylogenetic structure in functional traits, particularly those that emerge from the coordinated activity of multiple genes^24^. For example, bacterial traits such as growth rates in the presence of labile C show strong phylogenetic signals, whereas other traits such as responses to priming show shallow phylogenetic signals^22,23^. Thus, the level of phylogenetic resolution required to characterize variation in CUE across bacterial taxa remains unclear.

In addition to understanding the phylogenetic structure of variation in CUE, it may be useful to explore whether particular genomic traits predict CUE. For example, copy numbers of ribosomal RNA operons are inversely related to growth efficiency in bacteria, providing a method for predicting growth efficiencies from genomes^12^. Similarly, genomic traits have been useful for predicting microbial trophic strategies and biogeography, with bacterial taxa with larger genomes occupying a wider range of habitat types^25^ and dominating communities where resources are available in diverse forms but limiting concentrations^26^. Comparable efforts for predicting CUE from genomic traits are necessary to help overcome challenges with measuring taxa-specific CUE for highly diverse soil bacterial communities.

In the environment, microbial taxa are exposed to variations in substrate chemistry and supply rates that impact rates of C uptake and growth. These abiotic factors are likely to interact with intrinsic differences in physiology among taxa to ultimately determine CUE. For example, observations from bacterial cultures show that CUE increases with limiting resource concentration and with the free energy content of available resources^17^. These patterns are overlaid with differences between taxa, with potentially oligotrophic groups showing less responsivity to limiting resource availability than copiotrophic taxa^17^. Thus, estimates of CUE must consider both biotic and abiotic sources of variability, including bacterial physiology, substrate availability, and substrate chemistry.

Prior work on estimating CUE is limited to a small set of individual microbial taxa, or involves mixed, whole communities^10,11,17^. Direct measurements of CUE have been made using a wide range of methods including calorespirometry^11^ and stable isotope approaches^23,27^. CUE has also been estimated indirectly for whole communities based on environmental variables such as resource stoichiometry^10,28^. These methods can lead to CUE estimates that vary by a factor of two or more, making direct inter-comparisons challenging^10^.

Using a consistent methodology to measure CUE across a broad range of microbial taxa is necessary to determine how physiological variation in resource use between taxa impacts CUE. Metabolic models of bacterial physiology can be generated from annotated genomes^29^ and can be used to estimate taxa-specific biological fluxes, including biomass growth and C uptake^30^. Here, we explore an in silico approach to generate theoretical predictions of CUE for over 200 taxa using genome-scale constraint-based metabolic modeling. We find that intrinsic physiological differences between taxa can lead to >300% variation in CUE, which is far greater than that assumed in global models where CUE is either fixed or varies solely in relation to abiotic factors. We find that CUE is primarily structured at subphylum phylogenetic levels and is correlated negatively with genome size and GC content. These findings provide a framework for predicting CUE from genomic traits and for inferring potential impacts of shifts in bacterial community composition on C cycling. Using a recent ecosystem model of heterotrophic soil respiration (DAMM-MCNiP), we demonstrate that accounting for the observed variation in microbial physiology across taxa alone can have persistent implications for estimates of soil C emissions and soil C pool sizes.

## Results

### CUE from manually curated models varies by taxa and substrate

We first calculated CUE using the set of 13 manually curated, published metabolic models from diverse environments found in the BiGG database^31^. Flux balance analysis was performed for each metabolic model with C supplied exclusively through one of 14 individual C-containing metabolites, and CUE was calculated as the proportion of C assimilated into biomass relative to C uptake. We observe a mean CUE of 0.53 ± 0.25 (S.E.) across taxa and substrate types, suggesting that nearly half of consumed C is lost via respiration on average (Supplementary Table 1). However, these models also indicate wide variation in mean CUE between individual taxa (0.14 ± 0.07 to 0.84 ± 0.17, Supplementary Table 1) and equally large variation in mean CUE across substrate types (0.26 ± 0.24 to 0.66 ± 0.20, Supplementary Table 1).

### Potential CUE from draft models varies by taxa and substrate

**Fig. 1:**
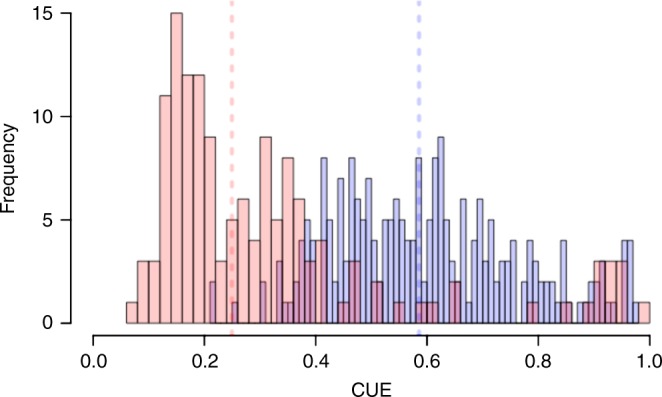
Potential and substrate-limited CUE. Histogram of predicted potential CUE (purple) and predicted CUE under lysine-limitation (pink) across taxa

Potential CUE values represent intrinsic variation in CUE based on genomic differences between taxa, and these values were most useful for comparisons between taxa and for identifying relationships between genome traits and CUE. Potential CUE ranges from 0.22 to 0.98 across all taxa, with a mean of 0.62 ± 0.17 (Fig. 1). The range of potential CUE values from this analysis corresponds to the high end of parameter settings currently used in microbial models of the C cycle (0.15–0.68).

**Fig. 2:**
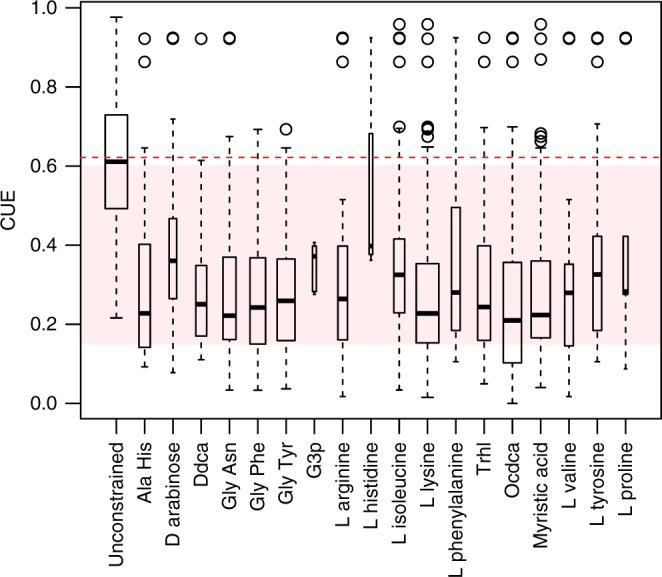
Substrate-limited CUE. Boxplot of average CUE values across all taxa under potential and constrained scenarios. Boxplot width is proportional to number of models with a given constraining reaction. Dashed red line shows average for potential CUE. Shaded region shows range of values typically used in biogeochemical models. Solid lines within boxplots show median. Bottom and top edges of boxes represent 25th and 75th percentiles, respectively. Whiskers demarcate minimum and maximum datapoints within 1.5× of the interquartile range

To assess the impact of substrate chemistry on CUE, we calculated the dependence of biomass production on all transport reactions associated with C uptake and secretion. We then identified the set of C-containing metabolites that most commonly limited biomass production across the full set of taxa in our analysis, and calculated CUE after reducing the availability of each of these constraining metabolites individually. The most common constraining reactions were related to amino acid and dipeptide uptake (Supplementary Table 2, Fig. 2). When uptake of individual constraining metabolites was set to reduce biomass production by 75%, mean CUE across all 18 constraining metabolites was 0.29 ± 0.19. This corresponds to an average decline in CUE of 0.33, or a 53% reduction in CUE, compared to the potential CUE scenario.

### Potential CUE is associated with genomic traits

**Fig. 3:**
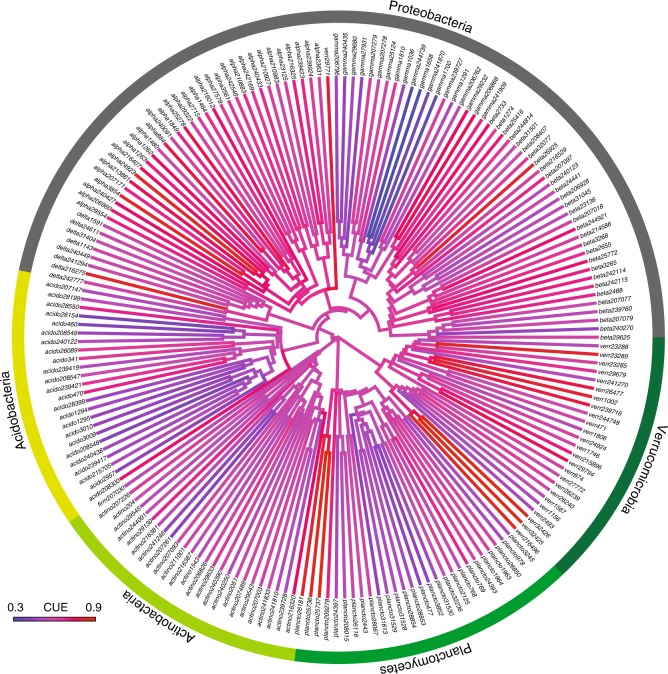
Phylogenetic heatmap of potential CUE. Phylogenetic heatmap of potential CUE values from draft metabolic models. Labels on tips correspond to kBase accession numbers

Potential CUE shows a significant phylogenetic signal (*K* = 0.99, *p* < 0.01, Fig. 3), indicating a Brownian pattern of trait evolution, with closely related taxa showing similarity in potential CUE values. The class (CI = 0.02 ± 0.019, Supplementary Table 3) and order (CI = 0.016 ± 0.020, Supplementary Table 3) levels explained the most variation in CUE. Therefore, these phylogenetic levels may be more appropriate than the phylum level for considering relationships between C cycling and bacterial community composition.

**Fig. 4:**
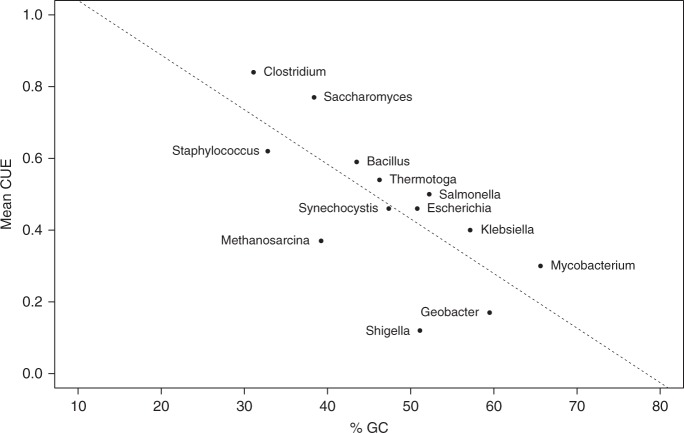
Mean CUE versus GC content. Mean CUE versus GC content for manually curated metabolic models. Species in order of increasing GC content are *Clostridium ljungdahlii DSM 13528*, *Staphylococcus aureus subsp. aureus N315*, *Saccharomyces cerevisiae S288c*, *Methanosarcina barkeri str. Fusaro*, *Bacillus subtilis subsp. subtilis str. 168*, *Thermotoga maritima MSB8*, *Synechocystis sp. PCC 6803*, *Escherichia coli str. K-12 substr. MG1655*, *Shigella boydii Sb227*, *Salmonella enterica subsp. enterica serovar Typhimurium str. LT2*, *Klebsiella pneumoniae subsp. pneumoniae MGH 78578*, *Geobacter metallireducens GS-15*, *Mycobacterium tuberculosis H37Rv*. Mean CUE was calculated from CUE on growth on each of the following C-sources individually: D-Glucose, Fumarate, Acetate, Acetaldehyde, 2-Oxoglutarate, Ethanol, Formate, D-Fructose, L-Glutamine, L-Glutamate, D-lactate, L-Malate, Pyruvate, Succinate

**Fig. 5:**
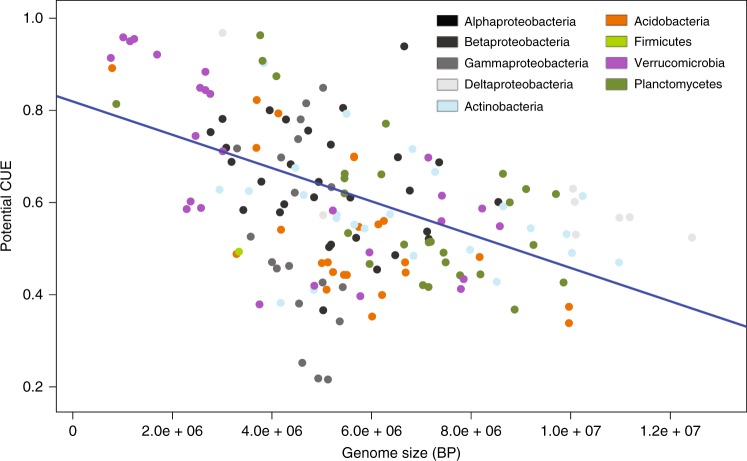
Potential CUE versus genome size. Potential CUE regressed against genome size (bp). Blue lines show GLS fit. Points are colored by phylum

Consistent with our observations based on the BIGG models (Fig. 4), we found a negative correlation between potential CUE and GC content in the larger set of metabolic models from kBase (Pseudo *R*^2^ = 0.20, Supplementary Table 4). In addition, potential CUE is significantly negatively correlated with genome size (Pseudo *R*^2^ = 0.36, Supplementary Table 4, Fig. 5), the number of genes coded for within a genome (Pseudo *R*^2^ = 0.34, Supplementary Table 4) and the number of transport reactions associated with C uptake and secretion (Pseudo *R*^2^ = 0.50, Supplementary Table 4).

### Variation in potential CUE impacts ecosystem-level C cycling

**Fig. 6:**
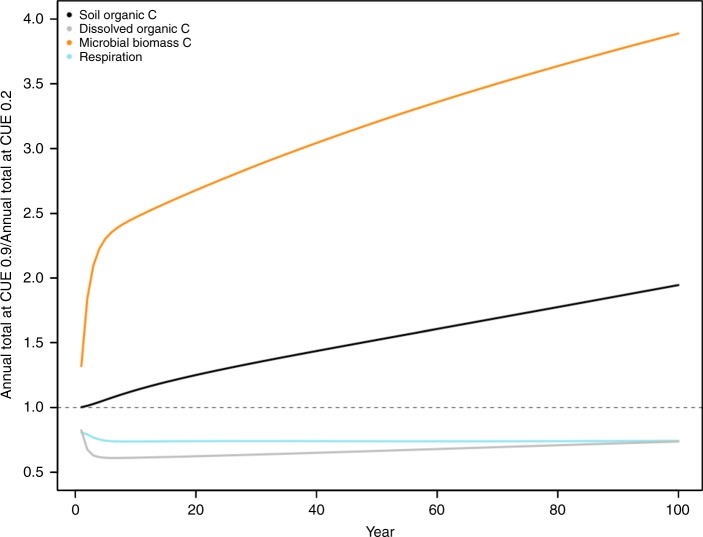
Ecosystem C stocks and fluxes with variable microbial communities. Annual totals for C cycle pools and respiration rates for models for high efficiency taxa (CUE = 0.9) relative to low efficiency taxa (CUE = 0.2) across 100 years. Dashed line represents no difference in model estimates at the two CUE values

Under a scenario in which the microbial community exhibited high efficiency (CUE = 0.9), soil organic C pool sizes were nearly twice as large following 100 years of simulation compared to the low efficiency scenario (CUE = 0.2, Fig. 6). This was driven, in part, by large sustained increases in microbial biomass, with the highly efficient microbial communities producing nearly four times greater microbial biomass than low efficiency communities over the same time span. Despite this large increase in microbial biomass, rates of respiration were reduced by 25% compared to the low efficiency communities (Fig. 6).

## Discussion

Genome-specific metabolic models have typically been used to explore variation in growth and microbial community interactions for small sets of microbial taxa^32,33^. To date, this approach has not been applied to better understand microbial CUE, a key parameter in emerging microbial models of the C cycle. Here, we show large, phylogenetically structured variation in potential CUE attributed to differences in physiology among >200 individual bacterial taxa. We observed that CUE was sensitive to substrate chemistry, substrate supply, and variation in microbial physiology between taxa. The intrinsic variation in CUE we observed among taxa is as large as that previously attributed to abiotic factors such as temperature and substrate chemistry^8,10^. For example, the temperature sensitivity of CUE for whole communities has been modeled as declining 0.4 units over a range of 25 ^o^C^5^, while we observed over 0.6 units of variation in potential CUE between individual bacterial taxa. We detected a significant phylogenetic signal in potential CUE corresponding to clustering at sub-phylum levels, and we found that potential CUE was negatively correlated with particular genome traits, including genome size and GC content. In addition, we identified a particular set of amino acids, dipeptides, fatty acids, and carbohydrates that resulted in large reductions in CUE when their availability was constrained. Finally, we found that the range of variation we observed in CUE across taxa could have major implications for estimates of respiration and C storage at the ecosystem level.

Overall, we observed a mean potential CUE of 0.62 ± 0.17 (Fig. 1), which may represent a mean maximum CUE for bacteria in the absence of resource limitation. Potential CUE values represent intrinsic variation in CUE based on genomic differences between taxa, and these values were most useful for comparisons between taxa and for identifying relationships between genome traits and CUE. The range of observed potential CUE values from this analysis corresponds to the high end of parameter settings currently used in microbial models of the C cycle (0.15–0.68). However, empirical measurements of CUE extend well above this mean in the absence of resource limitation^9^. When the availability of metabolites was constrained to reflect more reasonable expectations of resource limitation in the environment, we observed consistent declines of approximately 53% with CUE averaging 0.29 ± 0.19 (Supplementary Table 2).

Potential CUE varied across bacterial lineages, although not at the phylum-level. The significant phylogenetic signal in potential CUE indicates a Brownian pattern of trait evolution, with closely related taxa showing similarity in potential CUE values. However, we did not observe significant differences in potential CUE between bacterial phyla, and the greatest level of variation was structured at finer phylogenetic resolutions, including the class and order levels (Supplementary Table 3). Similar conclusions warning against broad phylum-level generalizations regarding carbon use traits have emerged from recent work using stable-isotope approaches^22^.

Certain genomic traits, such as GC content and genome size, can be useful predictors of bacterial niche preferences and the response of bacterial communities to environmental changes^12,25,34^. Bacteria with larger genomes must allocate greater resources towards maintenance, while smaller genomes can exhibit greater efficiency^35^. In soil, taxa with larger genomes tend to dominate communities where substrates are available in diverse forms but with limiting concentrations^26^, and bacterial taxa with larger genomes tend to occupy a wider range of habitat types^25^. In our analysis, potential CUE declined by 0.04 units per additional Mbp in a genome (Fig. 5). However, there was a tradeoff between efficiency and access to substrates, as taxa with larger genomes were able to access a larger breadth of C sources at the cost of reductions in potential CUE. Thus, taxa with the highest CUEs may be less adaptable to changes in substrate chemistry, representing a more specialized trophic strategy. Prior studies also observe copiotrophic taxa having large numbers of genes associated with transport proteins, which would correspond to large numbers of transport reactions associated with C uptake and secretion^34^.

Nutrient limitation can lead to shifts in community composition that favor GC-poor genomes, potentially due to the greater energetic cost of producing GTP and CTP bases^36^. Consistent with these findings, we observed a strong negative correlation between CUE and GC content (*R*^2^ = 0.522; Fig. 4). Thus, environmental changes that favor GC-poor genomes may also have ramifications for C cycling through correlated increases in CUE and corresponding reductions in CO_2_ emissions.

In the environment, microbial taxa are exposed to variation in substrate chemistry and availability, which can impact rates of C uptake and growth^37^. In prior studies, CUE shows sensitivity to substrate availability and stoichiometry at both the organismal^9,17^ and community levels^8,28^. In our analysis, we identified several specific amino acids and dipeptides whose availabilities limited CUE. These findings comport with patterns of amino acid uptake by bacteria in the environment^38,39^ and the incorporation of amino acids, such as alanine, directly into cell wall components. Amino acids represent a key input of N in soil^40^, and rapid uptake by microbes results in short residence time of these compounds in soil^41,42^. It is hypothesized that microbes consume amino acids primarily as a C source, which may support the large impact of constraining amino acid availability on CUE we observed. Similarly, dipeptides contain higher C:N ratios than their component amino acid monomers, and their uptake is greater than that of amino acids^43^.

Structured variability in soil organic matter, chemistry in soil could favor particular bacterial taxa over others based on their capacity to consume available C sources. In our analysis, taxa with the ability to consume a wide range of metabolites showed the lowest potential CUE values because of increased uptake of non-essential C-containing metabolites (Supplementary Table 4). In contrast, taxa with fewer exchange reactions were able to maintain higher CUE in the potential environment through reduced C uptake. These differences may be related to differences between copiotrophs and oligotrophs in terms of resource specialization, with less-specialized copiotrophic taxa showing lower CUE. Prior studies also observe copiotrophs having large numbers of genes associated with transport proteins, which would correspond to large numbers of C-containing exchange reactions in this analysis^34^.

Accounting for the variation in CUE, we observed across taxa can have significant consequences to ecosystem-level estimates of C pool sizes and respiration rates (Fig. 6). Soil organic C pool sizes were reduced by almost half when a community shift towards low efficiency bacteria (CUE = 0.2, Fig. 6) was modeled compared to a community comprised of high efficiency bacteria (CUE = 0.9). These values represent the extremes of our potential CUE observations and therefore represent the widest range of expected outcomes. The change in soil organic C pool sizes we observed was driven, in part, by large sustained increases in microbial biomass, with the highly efficient microbial communities producing nearly four times greater microbial biomass than low efficiency communities over the same time span. Despite this large increase in microbial biomass, rates of respiration were reduced by 25% compared to the low efficiency communities (Fig. 6). Thus, accounting for variation in CUE among taxa alone can have significant consequences to ecosystem-level estimates of C storage and respiration rates, and these differences can persist even at decadal timescales. While DAMM-MCNiP was parameterized and validated for a specific temperate forest ecosystem, the general model structure and its dependence on CUE are representative of soil C models used across several ecosystem types^44^, suggesting that variation in CUE across taxa is likely to have important implications for soil C cycling more broadly. In this analysis, only CUE was varied across model runs to isolate the impact of variation in microbial physiology while holding other parameter settings constant. However, shifts in microbial composition and physiology may also be concomitant with shifts in other parameters, such as those describing enzyme activities, making it critical to characterize these potential interacting effects to fully describe parameter uncertainty in the future.

Direct comparisons between the values observed here and those in other studies are challenging as potential CUE may not have exact parallels to empirical observations in which CUE has been measured for a small number of individual taxa or complex soil communities. We interpret potential CUE values as intrinsic variation based on genetic differences between taxa that may be most useful in terms of exploring comparisons between taxa and for identifying relationships between genome traits and CUE. Despite the challenges with measuring CUE, developing empirical approaches^11,23^ to directly estimate taxa-specific CUE will be necessary and useful for validating these observations.

It is important to note that the limited capacity of ascribing functions to genes through annotation pipelines, the poor knowledge of taxon-specific microbial biomass composition, and the need to implement a gap-filling algorithm to compensate for missing reactions in genome scale reconstructions can each impact flux estimates generated through FBA models. Despite these limitations, models generated through the pipeline used for our draft predictions have been demonstrated to closely match empirical phenotype data^32^, have been used to explore complex community interactions^45^, and have been shown to successfully predict community structure and environmental metabolomics^46^. We expect that future advancements in genome annotation and metabolic model construction paired with taxa-specific empirical observations of CUE could use the same conceptual framework proposed here to provide predictions with improved precision and fidelity. Collecting empirical measurements of taxa-specific bacterial CUE across a range of substrate types using a consistent methodology is recommended for further validation of these hypotheses.

The range of potential CUE values we observed between taxa is comparable to that observed in other studies in which wide ranges of CUE are attributed to differences between ecosystems or due to abiotic factors^10^. Soil microbial communities undergo shifts in composition under global change^47^, and these changes may alter the overall soil microbial CUE if particular taxa with uniquely high or low CUE values are favored based on growth strategy or substrate preference. Failing to account for relationships between CUE and microbial community composition may cause ecosystem models to miss important biotic feedbacks that can impact respiration fluxes and soil-C balance^6^. This analysis explores a method for generating draft predictions of taxa-specific CUE from metabolic models and identifies genome size and GC content as traits that may link genomic variation with C utilization strategies. We show that large phylogenetic variation in CUE between individual taxa make microbial physiology and community composition important factors to consider when estimating microbial contributions to C cycling.

## Methods

### Metabolic modeling

Genome-scale metabolic modeling (also known as stoichiometric modeling, or constraint-based modeling) can be used to quantitatively analyze the complete set of metabolic reactions in an organism. This approach has been successfully used to represent bacterial metabolism and growth patterns^32,48^, uptake and secretion^49,50^, and complex community interactions^33^ in silico. The metabolic model for a given organism can be generated by extracting the list of all biochemical reactions available to an organism from its annotated genome. In addition to intracellular reactions, the model includes exchange reactions, which involve uptake or secretion of metabolites, either through genome-encoded transporters, or expected free diffusion through the membrane. For convenience of subsequent mathematical analysis, this list is converted into a stoichiometric matrix, **S**, whose element *S*_*ij*_ corresponds to the stoichiometric coefficients of metabolite *i* in reaction *j*. Due to incomplete genome annotations, gapfilling is often required to supplement models with additional reactions before models are capable of producing a nonzero biomass flux.

Genome-scale metabolic models were selected for analysis from two separate databases. Thirteen microbial models were selected from the Biochemically, Genetically, and Genomically structured knowledgebase of metabolic reconstructions (BiGG), which contains a small set of manually curated metabolic models from diverse environments^31^. We utilized the following thirteen taxa in this analysis: *Clostridium ljungdahlii DSM 13528*, *Staphylococcus aureus subsp. aureus N315*, *Saccharomyces cerevisiae S288c*, *Methanosarcina barkeri str. Fusaro*, *Bacillus subtilis subsp. subtilis str. 168*, *Thermotoga maritima MSB8*, *Synechocystis sp. PCC 6803*, *Escherichia coli str. K-12 substr. MG1655*, *Shigella boydii Sb227*, *Salmonella enterica subsp. enterica serovar Typhimurium str. LT2*, *Klebsiella pneumoniae subsp. pneumoniae MGH 78578*, *Geobacter metallireducens GS-15*, and *Mycobacterium tuberculosis H37Rv*. Mean CUE was calculated from CUE on growth on each of the following C-sources individually: D-Glucose, Fumarate, Acetate, Acetaldehyde, 2-Oxoglutarate, Ethanol, Formate, D-Fructose, L-Glutamine, L-Glutamate, D-lactate, L-Malate, Pyruvate, Succinate. Due to the limited number of manually curated microbial metabolic models, we expanded our approach to include models generated using automated pipelines (described below) for over 200 bacterial taxa from phyla commonly observed in soil environments.

### Genome selection

The BiGG database primarily includes microbial models associated with the human microbiome, limiting our capacity to extrapolate our findings from these well-curated metabolic models to environmental microbial communities. We expanded our approach to target bacterial genomes belonging to phyla commonly observed in soil environments^19,47^, which are of particular interest due to their major contributions to global respiration. We queried the Department of Energy’s kBase for over 200 taxa and used automated pipelines to construct a large set of draft metabolic models.

The Department of Energy systems biology knowledgebase^51^ (kBase) was searched in March 2016 for bacterial genomes belonging to phyla that have been observed to dominate forest soil bacterial community composition based on 16S ribosomal RNA and DNA sequencing^15,47^. A total of 23,530 genomes belonging to the six selected phyla were identified in kBase, corresponding to 1064 unique genera. For each phylum, at least 25 genomes were selected for analysis. For phyla with more than 50 available genomes, the full list of unique genera was scanned to target genera that have been observed in soil environments when possible (Supplementary Fig. 2).

The Build Metabolic Model tool was used in kBase to generate metabolic models from 231 selected genomes^32^. Model construction in kBase involves functional annotation of the genome to identify metabolic genes and their associated biochemical reactions using the *Rapid Annotation* of microbial genomes using *Subsystems Technology* (RAST) genome annotation pipeline and the model SEED framework^32,52^. Draft metabolic models were gapfilled using the Gapfill Metabolic Model tool in kBase to add the minimal set of reactions required to produce biomass on complete media, which contains all possible metabolites available for uptake^53^. Gapfilling on complete media results in conservative gapfilling by assuming that metabolites necessary for growth but not produced intracellularly based on genome annotation are available in the environment.

### Flux balance analysis

Flux balance analysis (FBA) allows for an estimation of metabolic fluxes, such as rates of C uptake and utilization, through a metabolic model based on linear optimization of a specified objective function, such as biomass production. FBA makes a steady-state assumption, circumventing the need for knowledge of kinetic parameters, and uses the stoichiometry of metabolic reactions to determine the feasible space of all possible combinations of reaction rates. By prescribing an optimization scheme, it is possible to identify specific points in this feasible space, resulting in putative predictions of all metabolic reaction rates in the organism, including uptake and secretion fluxes and growth. This approach requires specification of (1) a flux or set of fluxes to maximize (or minimize) and (2) upper and lower bounds for all reactions within the metabolic model. Upon specification of these inputs, FBA is able to estimate the particular combination of fluxes through all reactions in the model that satisfy the given conditions. FBA was performed in MATLAB R2014a using the *optimizeCbModel* command in the *COnstraint-Based Reconstruction and Analysis* (COBRA) Toolbox^54^. All FBA analyses were set to maximize bacterial biomass production in this analysis, in accordance with standard FBA assumptions^33^.

### C use efficiency

C use efficiency (CUE) is calculated as the proportion of C retained in biomass relative to total C uptake (Eq. 1). For a metabolic model with *n* exchange reactions, and where *C* is equal to the number of C atoms taken up or secreted in a given reaction:

1 $${\mathrm{CUE = }}\frac{{\mathop {\sum }\nolimits_1^n {\mathrm{Uptake}}\;{\mathrm{flux}}_i \times C_i{\mathrm{ - }}\mathop {\sum }\nolimits_1^n {\mathrm{Secretion}}\;{\mathrm{flux}}_i \times C_i}}{{\mathop {\sum }\nolimits_1^n {\mathrm{Uptake}}\;{\mathrm{flux}}_i \times C_i}}$$

For the set of manually curated models, the availability of one of 14 individual C sources was manipulated, and CUE was calculated under exclusive uptake of each metabolite separately. For the larger set of models from kBase, CUE was explored under two scenarios. (1) potential CUE was calculated by allowing a model to utilize all exchange reactions present, and (2) constrained CUE was calculated by limiting the availability of a single C-containing metabolite relative to the availability of all other metabolites. Potential CUE was calculated to explore intrinsic metabolic variation in CUE, and these values were most useful for comparisons between taxa and for identifying relationships between genome traits and CUE. All exchange reactions present in a model were made available for uptake by allowing for a default maximum flux of 1000 mmol grDW^−1^ h^−1^, where grDW indicates the cellular biomass dry weight in grams. As CUE was calculated as a ratio of fluxes, values were not sensitive to the order of magnitude of maximum flux bounds as long as these were consistent across reactions. Certain models produced a respiration flux of 0 mmol grDW^−1^ h^−1^ and were excluded from subsequent analyses of CUE.

To calculate CUE under conditions of limited substrate availability, reactions in each metabolic model were first classified according to the following hierarchy: (1) exchange, (2) C-containing, (3) utilized when available, (4) essential to biomass production, and (5) constraining to biomass production (Supplementary Fig. 1). For a given model, all C-containing exchange reactions with a nonzero flux under potential conditions were classified as utilized. The maximum uptake flux for each individual utilized reaction was then set to 0 mmol grDW^−1^ h^−1^ and FBA was performed again to identify reactions that were essential for biomass production. Finally, maximum uptake for all essential reactions was individually set to 5% of the maximum uptake flux for all other metabolites (50 mmol grDW^−1^ h^−1^), and FBA was performed again to detect the impact of constraining particular essential reactions. Reactions that resulted in a reduction of the biomass flux by at least 5% were classified as constraining, meaning that the biomass production flux showed a direct response to the availability of metabolites dictated by these reactions.

Uptake fluxes for the most commonly occurring constraining reactions across all models were analyzed to determine the response of biomass production relative to availability for each metabolite (biomass/uptake). For 18 of the most commonly constraining reactions, the uptake flux corresponding to a 75% reduction in the biomass flux was identified for each model. For all models containing a given constraining reaction, FBA was performed after setting the maximum uptake flux for the constraining reaction to this reduced value while leaving all other exchange reaction fluxes potential. Constrained CUE was then calculated according to equation (1). Constrained CUE was compared to potential CUE for all models with a given constraining reaction using paired T-tests and Cohen’s D calculated from the *lsr* package^55^ in R Studio^56^.

### Model evaluation and empirical comparisons

To test the sensitivity of our results to the method of gapfilling, two parallel sets of models were constructed for each taxon. One set of models were gapfilled to achieve a minimum biomass flux of 0.1 mmol grDW^−1^ h^−1^ while a second set was more heavily gapfilled to achieve a (default) minimum biomass flux of 1000 mmol grDW^−1^ h^−1^. A total of 246 exchange reactions, including 211 C-containing exchange reactions, were observed across 231 models gapfilled to the lower biomass threshold. A total of 318 exchange reactions, including 279 C-containing exchange reactions, were observed across 231 models gapfilled to the higher biomass threshold. On average, models gapfilled to the higher biomass threshold had only 8 additional C-containing exchange reactions. Gapfilling intensity had a significant impact on subsequent calculations including CUE, but inter-model comparisons and rank order were not strongly affected by gapfilling intensity. Potential CUE values calculated from models gapfilled at the two intensities were strongly correlated (Pearson’s rank correlation coefficient = 0.7).

### Phylogenetic analyses

The Build Phylogenetic Tree tool was used in the DOE kBase to generate a phylogenetic tree for 220 of the 231 genomes analyzed based on 49 highly conserved clusters of orthologous group (COG) families and the FastTree maximum likelihood method^57^. Branch lengths were computed according to the Grafen method^58^ using the *compute.brlen* command in the *ape* package^59^ in R Studio. To test for phylogenetic signals, Blomberg’s K statistic^60^ was calculated using the *multiPhylosignal* function in the *Picante* package^61^ in R Studio. Mean differences in potential CUE between phyla were compared using phylogenetic ANOVA with the *phylANOVA* function in the *phytools* package^62^ in R Studio.

In order to determine the taxonomic level which best describes variation in potential CUE, we used Phylocom to calculate the contribution index (CI) for each of the 191 nodes in the bacterial phylogeny^63^. The CI indicates how much a particular node on the phylogeny accounts for the total variation in potential CUE^64^. After calculating the CI for all 191 nodes in our analysis, we classified the subset of nodes where collective contributions accounted 90% of the variation in potential CUE based on the taxonomic level at which descendent species diverged.

The relationship between potential CUE and (1) the number of exchange reactions, (2) the number of C-containing exchange reactions, (3) genome size, (4) guanine-cytosine (GC) content, and (5) number of genes was assessed using phylogenetic generalized least-squares regression with the *nlme* package^65^ in R Studio. The proportion of variance explained by each predictor was estimated using a pseudo *R*^2^ value designed for nonlinear regression^66^ using the *r.squaredLR* function in the *MuMIn* package^67^, which estimates the improvement of the fit model relative to a null model based on a likelihood ratio test. The *stepAIC* function in the *MASS* package^68^ was additionally used to determine the simplest regression model with multiple predictors.

### Ecosystem modeling

The Dual-Arrhenius Michaelis-Menten Microbial C and Nitrogen Physiology (DAMM-MCNiP) model was used to estimate potential impacts of the observed variation in CUE on ecosystem-level C fluxes. DAMM-MCNiP models the effects of soil moisture and temperature on coupled C and nitrogen fluxes through soil pools and microbial biomass^69^. Specifically, the model uses Michaelis-Menten kinetics to describe the depolymerization of soil organic C and soil organic Nitrogen by microbial extracellular enzymes to produce dissolved organic C (DOC) and dissolved organic N (DON). Maximum reaction velocities are governed by temperature-sensitive Arrhenius functions. Uptake of DOC and DON by microbial biomass is governed by a second series of Arrhenius and Michaelis-Menten kinetic equations, which are sensitive to moisture-mediated O_2_ availability. Following uptake, the parameter CUE is used to determine the partitioning of C between microbial biomass and soil respiration. DAMM-MCNiP has been parameterized to describe seasonal patterns in heterotrophic soil respiration at a temperate forest site, and is able to capture 56% of variation in empirical observations of seasonal heterotrophic respiration at an hourly scale^69^ (RMSE = 0.25, *R*^2^ adjusted = 56). The published model uses a default CUE value of 0.3 as used in several other ecosystem models. We modified the parameterization of CUE in this model (while retaining all other parameter settings as described in detail in Abramoff et al. 2017, Supplementary Table 5) to reflect the range of variation observed in CUE across taxa. We then quantified the impact of this variation on model estimates over 100 repeated cycles of annual variation in temperature and moisture to assess long-term impacts.

### Reporting summary

Further information on research design is available in the Nature Research Reporting Summary linked to this article.

## Supplementary information

Supplementary Information

Reporting Summary

## Data Availability

Data used in this analysis are available in the supplementary material and additionally available upon request.
