# Supplementary material for: Microbial carbon use efficiency predicted from genome-scale metabolic models
Source: Nat Commun. 2019 Aug 8;10:3568. doi: 10.1038/s41467-019-11488-z (PMC6687798; doi:10.1038/s41467-019-11488-z)
Supplement: Supplementary file 1 — Supplementary Information [file 41467_2019_11488_MOESM1_ESM.pdf]

Supplementary Information

Microbial carbon use efficiency predicted from genome-scale metabolic models

Authors: Saifuddin, M. et al.

Supplementary Table 1 — CUE for BiGG models across substrate types.

Genus	Species	Genome Size (Mbp)	GC Content	D-Glucose	Fumarate	Acetate	Acetaldehyde	2-Oxoglutarate	Ethanol	Formate	D-Fructose	L-Glutamine	L-Glutamate	D-lactate	L-Malate	Pyruvate	Succinate
**Bacillus**	*subtilis subsp. subtilis str. 168*	4.22	43.50	0.94	0.70	NG	NA	0.64	NG	NG	0.94	0.64	0.64	0.94	0.70	0.78	0.70
**Clostridium**	*ljungdahlii DSM 13528*	4.63	31.10	0.95	NA	NA	NA	NA	NA	0.48	0.94	0.88	0.87	0.97	NA	0.80	NA
**Escherichia**	*coli str. K-12 substr. MG1655*	4.64	50.79	0.62	0.39	0.37	0.60	0.45	0.70	NG	0.62	0.48	0.51	0.50	0.39	0.41	0.42
**Geobacter**	*metallireducens GS-15*	4.01	59.49	NA	NA	0.11	NA	NA	0.28	0.06	NA	NA	NA	NA	NA	0.23	NA
**Klebsiella**	*pneumoniae subsp. pneumoniae MGH 78578*	5.69	57.15	0.63	NG	0.46	0.68	NG	0.80	NG	0.63	0.53	0.53	0.50	0.43	0.45	NG
**Methanosarcina**	*barkeri str. Fusaro*	4.87	39.23	NA	NA	0.55	NA	NA	NA	NA	NA	NA	NG	NA	NA	0.56	NA
**Mycobacterium**	*tuberculosis H37Rv*	4.41	65.61	0.43	0.25	NA	NA	NA	NA	NA	NA	NA	0.28	0.31	0.25	0.27	0.29
**Saccharomyces**	*cerevisiae S288c*	12.2	38.38	0.70	0.49	0.90	0.98	0.82	0.98	0.54	0.70	0.95	0.95	0.93	0.49	0.53	0.85
**Salmonella**	*enterica subsp. enterica serovar Typhimurium str. LT2*	4.81	52.23	0.64	0.44	0.44	0.66	0.48	0.78	0.02	0.64	0.55	0.54	0.50	0.44	0.45	0.48
**Shigella**	*boydii Sb227*	4.65	51.11	0.27	0.08	NG	0.14	0.15	0.14	NG	0.27	0.15	0.15	0.09	0.08	0.09	0.08
**Staphylococcus**	*aureus subsp. aureus N315*	2.84	32.81	0.60	NA	NA	NA	0.77	0.74	0.22	0.60	NA	0.56	0.54	0.94	NA	NA
**Synechocystis**	*sp. PCC 6803*	3.95	47.36	0.50	0.35	0.31	NA	0.36	NA	NA	0.50	0.81	0.62	NA	0.35	0.37	0.39
**Thermotoga**	*maritima MSB8*	1.86	46.25	0.72	NA	NG	NA	NA	NA	NA	0.72	NA	NA	0.70	NA	NA	NA

CUE from manually-curated metabolic models from BIGG database.
NG = no growth, NA = exchange reaction absent from model.

Supplementary Table 2 — CUE under substrate limitation

Constraining metabolite	Class	Mean ± SD Constrained CUE	Biomass/ Uptake Slope	Cohen's D	Paired T-test P-value
L-Lysine	Amino Acid	0.27 ± 0.17	3.25 ± 0.36	2.9	***
Myristic Acid	Fatty Acid	0.28 ± 0.17	6.48 ± 0.34	2.9	***
Gly-Phe	Dipeptide	0.27 ± 0.15	15.93 ± 15.53	2.8	***
Trehalose (trhl)	Carbohydrate	0.30 ± 0.19	4.81 ± 4.4	2.7	***
Gly-Tyr	Dipeptide	0.28 ± 0.15	8.52 ± 1.48	2.7	***
Glyceraldehyde 3 Phosphate (g3p)	Carbohydrate	0.35 ± 0.06	14.88 ± 12.33	2.7	**
Gly-Asn	Dipeptide	0.28 ± 0.18	5.25 ± 1.09	2.5	***
Stearic Acid (ocdca)	Fatty Acid	0.25 ± 0.20	8.38 ± 4.13	2.4	***
Lauric Acid (ddca)	Fatty Acid	0.28 ± 0.16	8.68 ± 4.66	2.4	***
Ala-His	Dipeptide	0.29 ± 0.20	12.79 ± 1.55	2.3	***
L-Valine	Amino Acid	0.30 ± 0.21	2.92 ± 0.28	2.2	***
L-Tyrosine	Amino Acid	0.37 ± 0.24	8.42 ± 0.28	1.9	***
L-Arginine	Amino Acid	0.32 ± 0.22	3.99 ± 0.76	1.9	***
L-Phenylalanine	Amino Acid	0.38 ± 0.24	6.43 ± 0.21	1.8	***
L-Isoleucine	Amino Acid	0.36 ± 0.23	3.21 ± 0.55	1.8	***
D-Arabinose	Carbohydrate	0.40 ± 0.22	13.33 ± 0	1.7	***
L-Proline	Amino Acid	0.41 ± 0.30	5.2 ± 1.63	1.6	**
L-Histidine	Amino Acid	0.55 ± 0.25	15.52 ± 6.49	1.3	*

Effect of constraining the availability of particular metabolites on CUE. Maximum uptake of constraining metabolite was set to reduce biomass to 25% of maximum flux, based on observed linear relationships between uptake and biomass production. Biomass/uptake slope indicates mean ± standard deviation of biomass flux per unit uptake flux for all models with a particular constraining metabolit. Cohen's D value compares potential CUE to constrained CUE for all models that have an exchange reaction for the given metabolite. P-values are from paired t-tests

comparing constrained and potential CUE for all models that have an exchange reaction for the given metabolite (***$P<0.001$,** $P<0.01$, * $P<0.05$).

Supplementary Table 3 — Contribution indices for variation in potential CUE by taxonomic level.

	Average CI	SD	Total CI
Class	0.020	0.019	0.299
Family	0.012	0.007	0.106
Genus	0.009	0.006	0.132
Order	0.016	0.020	0.286
Phylum	0.016	0.009	0.047
Species	0.010	NA	0.010
Strain	0.011	0.005	0.021

Contribution Index (CI) values for nodes accounting for 90% of variation in potential CUE.

Supplementary Table 4 — Regression summary for predictors of potential CUE.

	GLS intercept	GLS Slope	GLS P-Val	Pseudo R^2
Exchange reactions	1.187	-5.91×10^{-3}	<0.001	0.500
C-containing exchange reactions	1.066	-6.37×10^{-3}	<0.001	0.496
GC content	0.909	-4.86×10^{-3}	<0.01	0.201
Genes	0.822	-4.06×10^{-5}	<0.001	0.341
Genome size	0.819	-3.61×10^{-8}	<0.001	0.356

PGLS regression results for potential CUE regressed against individual predictors listed in rows. For multi-regression models, lowest AIC models included genome size, number of C-containing exchange reactions, and number of genes.

Supplementary Table 5

Parameter	Units	Value	Description
Ea_{dep}	kJ mol^{-1}	61.77	Activation energy for SOCN depolymerization
Km_{dep}	mg cm^{-3}	0.0025	Half-saturation constant for SOCN depolymerization
a_{dep}	mg SOCN cm^{-3} (mg Enz $cm^{-3})^{-1}$ h^{-1}	1.0815×10^{11}	Pre-exponential constant for SOCN depolymerization
Ea_{upt}	kJ mol^{-1}	61.77	Activation energy for DOC uptake
a_{upt}	mg DOCN cm^{-3} (mg biomass $cm^{-3})^{-1}$ h^{-1}	1.0815×10^{11}	Pre-exponential constant for DOCN uptake
Km_{upt}	mg cm^{-3}	0.3	Half-saturation constant for DOCN uptake
Km_{O2}	cm^3 O_2 cm^{-3} air	0.121	Michaelis constant for O_2
CUE	mg mg^{-1}	0.2–0.9	Carbon use efficiency
p	-	0.5	proportion of assimilated C allocated to enzyme production
q	-	0.5	proportion of assimilated N allocated to enzyme production
CN_s	-	27.6	C:N of soil
CN_l	-	27.6	C:N of litter
CN_m	-	10	C:N of microbial biomass
CN_e	-	3	C:N of enzymes
BD	g cm^{-3}	0.8	bulk density
PD	g cm^{-3}	2.52	particle density
r_death	hr^{-1}	0.00015	microbial turnover rate
r_EnzLoss	hr^{-1}	0.001	enzyme turnover rate

**MICtoSOCN**	mg mg^{-1}	0.5	fraction of dead microbial biomass allocated to SOCN
**a**	-	0.5	proportion of enzyme pool acting on SOC pool (1-a = proportion acting on SON pool)
**frac**	g C cm^{-3}/ g C cm^{-3}	0.000414	fraction of unprotected SOCN, using soluble substrate estimated from Magill et al., 2000
**sat**	cm^3 H_2O cm^{-3} soil	1	Moisture saturation level
**$O_{2airfrac}$**	L O_2/ L air	0.209	volume fraction of O_2 air
**D_{liq}**	-	3.17	diffusion coefficient for unprotected SOCN and DOCN in liquid
**D_{gas}**	-	1.67	diffusion coefficient for O_2 in air

Supplementary Figure 1 — Metabolic model reaction classification framework

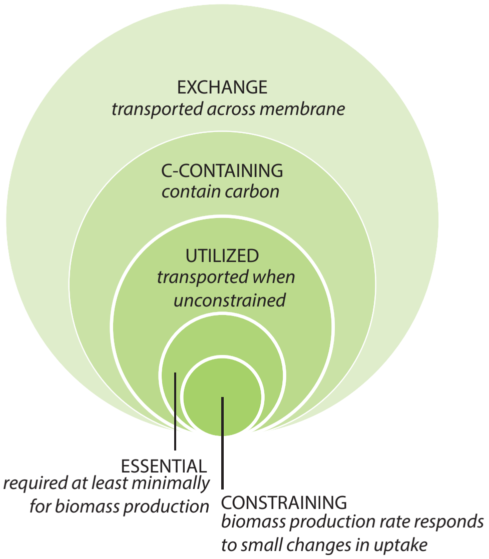

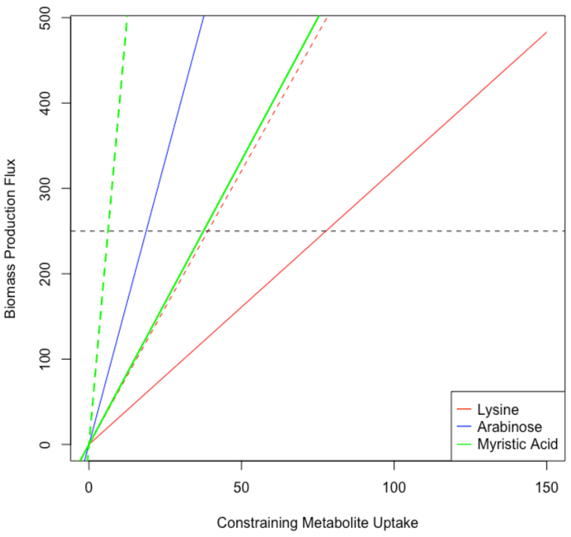

(A) Framework for classifying reactions to identify metabolites impacting CUE. (B) Uptake fluxes for all common constraining metabolites were analyzed. Fluxes for three metabolites and selected taxa are shown as an example, with lines colored by metabolite. Solid red line shows biomass response to L-lysine uptake seen for 186 models, including *Terriglobus saanensis SP1PR4* and *Starkeya novella DSM 506*. Dashed red line shows biomass response to L-lysine uptake for *Verrucomicrobia bacterium SCGC AAA164-I21*. Blue line shows biomass response to D-Arabinose uptake seen for 23 models, including *Verrucomicrobia bacterium SCGC AAA164-I21*. Solid green line shows biomass response to myristic acid uptake seen for 106 models including *Terriglobus saanensis SP1PR4.* Dashed green line shows biomass response to myristic acid uptake seen for 38 models including *Starkeya novella DSM 506.* Uptake values corresponding to 25% maximum biomass production (dashed black line) were calculated for each of the most common constraining metabolites for each taxa. This uptake value was then used as the maximum uptake flux for constrained CUE calculations.

Supplementary Figure 2

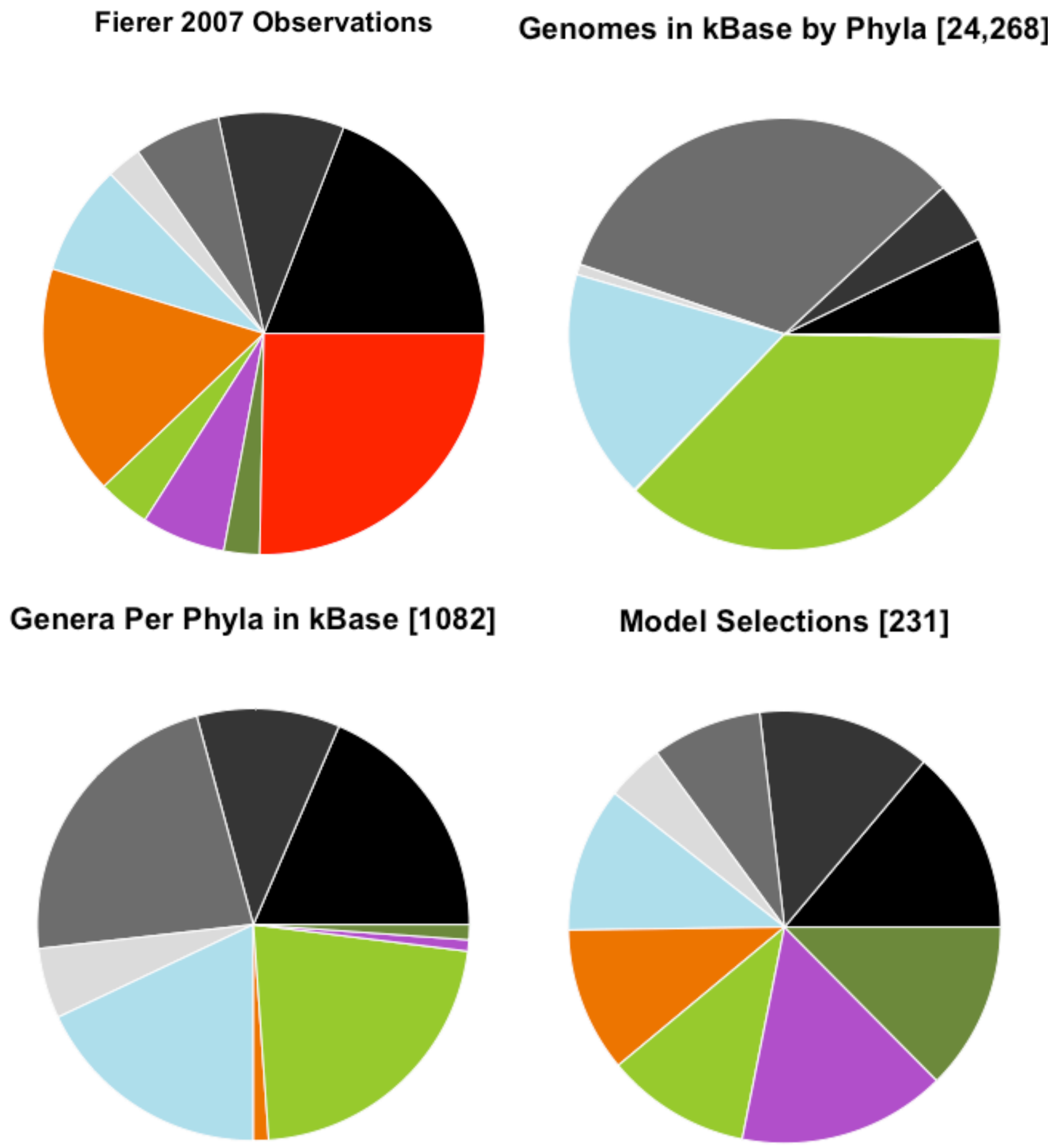

(A) Observations of dominant taxa in bulk soil microbial communities from meta-analysis of published clone libraries from diverse environments (adapted from Fierer *et al.* 2007). (B) Summary of genomes available in kBase based on phyla of interest. (C) Number of unique genera per phylum available in kbase. (D) Subset of genomes selected for metabolic modeling analysis.
