# Supplementary material for: Microbial carbon use efficiency predicted from genome-scale metabolic models
Source: Nat Commun. 2019 Aug 8;10:3568. doi: 10.1038/s41467-019-11488-z (PMC6687798; doi:10.1038/s41467-019-11488-z)
Supplement: Supplementary file 2 — Reporting Summary [file 41467_2019_11488_MOESM2_ESM.pdf]

nature research

Corresponding author(s): Adrien Finzi (afinzi@bu.edu)

Reporting Summary

Nature Research wishes to improve the reproducibility of the work that we publish. This form provides structure for consistency and transparency in reporting. For further information on Nature Research policies, see Authors & Referees and the Editorial Policy Checklist.

Statistical parameters

When statistical analyses are reported, confirm that the following items are present in the relevant location (e.g. figure legend, table legend, main text, or Methods section).

n/a	Confirmed	
☐	☒	The exact sample size (n) for each experimental group/condition, given as a discrete number and unit of measurement
☐	☒	An indication of whether measurements were taken from distinct samples or whether the same sample was measured repeatedly
☐	☒	The statistical test(s) used AND whether they are one- or two-sided<br>*Only common tests should be described solely by name; describe more complex techniques in the Methods section.*
☐	☒	A description of all covariates tested
☐	☒	A description of any assumptions or corrections, such as tests of normality and adjustment for multiple comparisons
☐	☒	A full description of the statistics including central tendency (e.g. means) or other basic estimates (e.g. regression coefficient) AND variation (e.g. standard deviation) or associated estimates of uncertainty (e.g. confidence intervals)
☐	☒	For null hypothesis testing, the test statistic (e.g. F, t, r) with confidence intervals, effect sizes, degrees of freedom and P value noted<br>*Give P values as exact values whenever suitable.*
☒	☐	For Bayesian analysis, information on the choice of priors and Markov chain Monte Carlo settings
☒	☐	For hierarchical and complex designs, identification of the appropriate level for tests and full reporting of outcomes
☐	☒	Estimates of effect sizes (e.g. Cohen's d, Pearson's r), indicating how they were calculated
☐	☒	Clearly defined error bars<br>*State explicitly what error bars represent (e.g. SD, SE, CI)*

Our web collection on statistics for biologists may be useful.

Software and code

Policy information about availability of computer code

Data collection	Genomes were selected from the Department of Energy's Knowledgebase (Kbase, https://kbase.us/) and metabolic models were generated using publicly-available tools on the kbase platform. The full list of selected genomes and their associated Kbase IDs is presented in the supplementary information. A separate set of curated metabolic models from the BiGG database (http://bigg.ucsd.edu) was also utilized, and full taxa names for these are provided in the associated figure legend. For all models, flux balance analysis was performed using the open-source COnstraint-Based Reconstruction and Analysis (COBRA) Toolbox in MATLAB R2014a. We also assembled empirical observations of CUE from literature using Web of Science.
Data analysis	Calculations of CUE and subsequent phylogenetic and statistical analyses were conducted in R Studio using the cited, publicly available statistical packages.

For manuscripts utilizing custom algorithms or software that are central to the research but not yet described in published literature, software must be made available to editors/reviewers upon request. We strongly encourage code deposition in a community repository (e.g. GitHub). See the Nature Research guidelines for submitting code & software for further information.

Data

Policy information about availability of data

All manuscripts must include a data availability statement. This statement should provide the following information, where applicable:

- Accession codes, unique identifiers, or web links for publicly available datasets
- A list of figures that have associated raw data
- A description of any restrictions on data availability

Data used in this analysis are available in the supplementary material and additionally available upon request.

Field-specific reporting

Please select the best fit for your research. If you are not sure, read the appropriate sections before making your selection.

☐ Life sciences ☐ Behavioural & social sciences ☒ Ecological, evolutionary & environmental sciences

For a reference copy of the document with all sections, see nature.com/authors/policies/ReportingSummary-flat.pdf

Ecological, evolutionary & environmental sciences study design

All studies must disclose on these points even when the disclosure is negative.

Study description	Genome-scale metabolic models were utilized (1) to estimate bacterial carbon use efficiency (CUE), (2) to determine how CUE varies phylogenetically and in relation to substrate chemistry, and (3) to determine the potential ecosystem-level impact of phylogenetic variation in CUE on carbon cycling.
Research sample	Individual metabolic models were treated as samples in this study. This includes 200+ bacterial species with genomes accessed from the Department of Energy's knowledgebase as well as 13 microbial metabolic models accessed from the BiGG database.
Sampling strategy	Genomes were selected by first searching the Department of Energy's knowledgebase for taxa belonging to specific phyla commonly found in soil environments. For each targeted phylum, we sought to access at least 25 individual representative bacterial taxa. For phyla with more than 50 available genomes, the full list of unique genera within the phylum was scanned to target genera that have been observed in soil environments. Phylogenetic-relatedness was accounted for in our statistical analyses. Sampling from the BiGG database followed a similar approach, in which we selected individual models belonging to different microbial genera, representing almost the full range of unique genera available in this database.
Data collection	Flux balance analysis was performed using the open-source COnstraint-Based Reconstruction and Analysis (COBRA) Toolbox in MATLAB R2014a. Carbon Use Efficiency was calculated based on the equations described in the the methods section.
Timing and spatial scale	All metabolic models were generated and downloaded from the DOE kBase in March 2016.
Data exclusions	Certain metabolic models showed growth with zero respiration fluxes and were excluded from this analysis as they did not correspond to our defined expectations for calculating carbon use efficiency.
Reproducibility	Performing flux balance analysis on the downloaded set of metabolic models results in consistent, reproducible estimates of CUE.
Randomization	Randomization was not relevant to this study as all analyses were performed in silico.
Blinding	Blinding was not relevant to this study as all analyses were performed in silico.

Did the study involve field work? ☐ Yes ☒ No

Reporting for specific materials, systems and methods

Materials & experimental systems

n/a	Involved in the study
☒	☐ Unique biological materials
☒	☐ Antibodies
☒	☐ Eukaryotic cell lines
☒	☐ Palaeontology
☒	☐ Animals and other organisms
☒	☐ Human research participants

Methods

n/a	Involved in the study
☒	☐ ChIP-seq
☒	☐ Flow cytometry
☒	☐ MRI-based neuroimaging
